# A large-scale crop protection bioassay data set

**DOI:** 10.1038/sdata.2015.32

**Published:** 2015-07-07

**Authors:** Anna Gaulton, Namrata Kale, Gerard J. P. van Westen, Louisa J. Bellis, A. Patrícia Bento, Mark Davies, Anne Hersey, George Papadatos, Mark Forster, Philip Wege, John P. Overington

**Affiliations:** 1 European Molecular Biology Laboratory —European Bioinformatics Institute, Wellcome Trust Genome Campus, Hinxton, Cambridgeshire CB10 1SD, UK; 2 Syngenta, Jealott’s Hill International Research Centre, Bracknell, Berkshire RG42 6EY, UK

**Keywords:** Chemical biology, Plant sciences, Agroecology, Microbiology

## Abstract

ChEMBL is a large-scale drug discovery database containing bioactivity information primarily extracted from scientific literature. Due to the medicinal chemistry focus of the journals from which data are extracted, the data are currently of most direct value in the field of human health research. However, many of the scientific use-cases for the current data set are equally applicable in other fields, such as crop protection research: for example, identification of chemical scaffolds active against a particular target or endpoint, the de-convolution of the potential targets of a phenotypic assay, or the potential targets/pathways for safety liabilities. In order to broaden the applicability of the ChEMBL database and allow more widespread use in crop protection research, an extensive data set of bioactivity data of insecticidal, fungicidal and herbicidal compounds and assays was collated and added to the database.

## Background & Summary

ChEMBL (https://www.ebi.ac.uk/chembl/) is a large-scale drug discovery database containing information about bioactive molecules, their interaction with molecular targets and their biological effects^1,2^. These data are manually extracted from full-text scientific articles in peer-reviewed medicinal chemistry journals and include information about the compounds synthesized or tested (together with their 2D chemical structures), the assays performed on these compounds and the molecular targets of those assays (where known). All experimental activity measurements are captured from the articles, regardless of whether these are binding affinity measurements against protein targets, phenotypic outcomes in whole organism assays or measurements of pharmacokinetic or physicochemical parameters. The most recent release of the database contains over 1.4 million compounds and 13.5 million activity data points and therefore provides a rich resource for addressing a wide range of drug-discovery questions. Examples of the utility of ChEMBL include investigation of rules for lead optimization, such as identification of bioisostere replacements or activity cliffs^3,4^; training models for prediction of the likely targets of a compound^5,6^, and subsequent use in de-convoluting phenotypic assays^7^; assessing druggability and drug properties, and prioritizing targets on a genome-wide scale^8,9^; and as a core component of a number of other resources and data integration platforms^10,11^.

While the existing ChEMBL data set has thus far mainly found applications in human health, due to the focus of the journals from which it has been extracted, this type of data set can have similar applicability in other areas of life science, such as crop protection research. Though many of the chemotypes, molecular targets and species involved may differ in this field, the broad applications of a large-scale bioactivity data set remain equally relevant. We therefore sought to supplement the data currently in ChEMBL with a rich set of crop protection bioactivity data. A number of key journals were identified as being of particular interest for data extraction, and a text-mining approach^12^ was also used to identify additional articles within PubMed that were likely to contain herbicidal, fungicidal or insecticidal bioactivity data. Compound structures, assay information and activity measurements were extracted from these articles, and the extracted data were further curated to standardize chemical structures, normalize assay descriptions and species names and identify molecular targets. Finally, in order to allow the broadest applicability of the new data set, the information was integrated into the ChEMBL database (version 19), allowing crop protection data to be viewed and analysed along side human health-related information.

## Methods

### Content identification

Publications containing relevant data were selected in two ways. Firstly a set of documents was selected using the ChEMBL-likeness text-mining algorithm, which has been published previously^12^. The ChEMBL-likeness algorithm was trained on the ChEMBL_15 corpus and an equally sized set of random MedLine abstracts that were not in ChEMBL. 141,252 abstracts containing crop protection-related keywords (see Supplementary File 1) were retrieved from MedLine and scored using the algorithm. Additional factors such as the availability of Open Access and access costs for the papers were also considered. The top 600 articles identified by this process were kept for abstraction. Secondly, four journals were identified as having significant crop protection content (Medicinal Chemistry Research, Crop Protection, Pest Management Science and Journal of Agricultural and Food Chemistry). All papers containing bioactivity data were therefore extracted from these journals.

The list of articles resulting from this selection process is shown in Supplementary Table 1.

### Data extraction

Data were manually extracted from full-text of selected articles, following a set of curation guidelines, and were supplied according to the ChEMBL deposition template (ftp://ftp.ebi.ac.uk/pub/databases/chembl/ChEMBLNTD/ChEMBL_Deposition_Template.tar.gz). For each extracted article, full citation details were provided, including either a PubMed ID or DOI. All reported compounds that had been tested for activity measurements (including qualitative measurements and negative results) were drawn in full, including any salt if present, and stored as MDL Molfiles^13^. Compound names as recorded in the original articles were also extracted. All of the performed assays (including binding, functional/phenotypic, toxicity and physicochemical property assays) were recorded with a succinct but meaningful description of the experiment, further annotated with information on the species, strain, tissue, cell line or subcellular fraction used, and the name and/or UniProt identifiers of targets, where known. All measurements reported for each compound/assay were extracted together with their units and any qualifier used (e.g., =, >, <, <=). Qualitative measurements (e.g., ‘Inactive’, ‘Not toxic’) were also extracted and recorded as an activity comment.

### Data standardization and curation

A purpose-built Pipeline Pilot^14^ protocol was used to standardize compound structures according to the established ChEMBL business rules^2^, which are based on the FDA substance registration system guidelines^15^. This included Kekulization of aromatic bonds; correction of incorrect valences; standardization of nitro and sulfoxide groups, steroid stereochemistry and halide salts; protonation/deprotonation of acids/bases to produce neutral molecules wherever possible; and moving stereochemistry from ring bonds onto adjacent hydrogen atoms. In addition ‘parent’ molecules were also generated, by removing isotope and salt information, so that bioactivity data could be grouped at the parent level whilst still recording the molecular form used in the experiment. For parent molecules a range of properties such as ALogP, molecular weight, number of hydrogen bond donors/acceptors, polar surface area and most acidic/basic pKa were calculated using Pipeline Pilot and ACDlabs Physchem software^16^. Compounds were integrated with existing ChEMBL compounds using the Standard InChI^17^ to determine identity, *i.e.,* compounds with a novel Standard InChI were registered as new compounds in ChEMBL, while those matching an existing Standard InChI were mapped to the existing entry in the ChEMBL molecule_dictionary. In order to generate cross-references to other compound-based resources (e.g., PubChem^18^, ZINC^19^, ChEBI^20^), the extracted compounds were also incorporated in the UniChem system^21,22^, as part of the ChEMBL data source.

Known pesticides were also assigned a mechanism of action classification according to the Fungicide Resistance Action Committee (FRAC), Herbicide Resistance Action Committee (HRAC) or Insecticide Resistance Action Committee (IRAC) systems^23–25^.

Assay descriptions were manually inspected to ensure accuracy and consistency of the details provided and to check the validity of the tissue, target and species names. Species were standardized to the taxonomy IDs and names used by the NCBI Taxonomy database^26^ and strain information was recorded separately. Each assay was assigned a BioAssay Ontology assay format term (e.g., biochemical, cell-based, tissue-based, organism-based) using a custom rule-based text classifier, based on the information provided by the assay description, and associated assay and target fields^27^. Cell-lines used in assays were also mapped to the ChEMBL cell_dictionary and to several other external ontologies: CLO^28^, EFO^29^ and Cellosaurus^30^.

Protein targets were mapped to corresponding primary accessions in the UniProt database^31^. Where a protein from the correct species was not available in UniProt, a close orthologue was selected and the ChEMBL relationship_type flag was used to record this. Where bioactivity was measured against a protein complex, the ChEMBL target recorded reflected this, and was mapped to the UniProt accessions for each of the individual protein subunits. Where molecular targets of assays were not known, tissue- or organism-level targets were assigned to the assay. Where the target assigned to an assay matched an existing ChEMBL target in both species and identity (according to the sequence and/or accession for a single protein, or sequence and/or accession of all target components for a protein complex) this target identifier was used, otherwise a new ChEMBL target was created with the appropriate type (e.g., SINGLE PROTEIN, PROTEIN COMPLEX, ORGANISM). The resulting entries in the ChEMBL target_dictionary were manually checked for redundancy, and any occurrences of multiple entries for the same protein were removed (for example many proteins have multiple UniProt entries with different sequences recorded due to errors, variants or partial sequences). For new targets, cross-references to other protein-based resources (e.g., Gene Ontology^32^, InterPro^33^, Pfam^34^) were generated from the cross-references provided by the corresponding UniProt entries.

The species tested in the data set were manually classified according to the standard ChEMBL organism classification system. To address the specific needs of this data set, an enhanced version of this classification, with greater focus on plants, arthropods and fungi, was also prepared and made available for download as an additional file. Proteins were also classified according to the ChEMBL protein family classification system (which covers key drug-discovery relevant protein families using community-defined and accepted classification schemes^35–39^).

Activity measurements were standardized according to the standard ChEMBL procedure described previously^1^ to ensure that common activity types (e.g., IC50, GI50, etc.) were provided with comparable units, and to flag potential erroneous measurements (e.g., possible transcription errors). Activity types were also mapped to BioAssay Ontology result terms and units to Unit Ontology^40^ and Quantities, Units, Dimensions and Data Types Ontologies (QUDT)^41^ terms.

**Figure 1:**
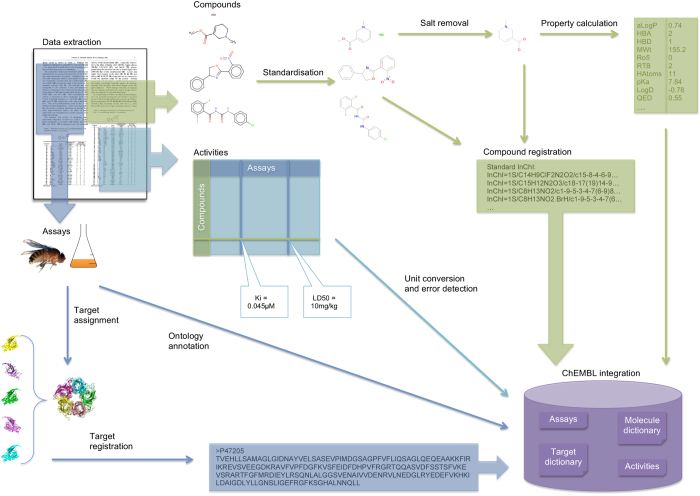
Diagram showing the data collection, standardization and integration process. Details of assays performed, compounds tested and activity measurements were extracted from full text publications. Data were further standardized to normalize compound structures, convert units of measurement and assign target information, before being integrated into the ChEMBL database.

Following curation and standardization, data were integrated into version 19 of the ChEMBL database (released 23rd July 2014). Fig. 1 shows an overview of the data extraction and standardization process.

## Data Records

A total of 2,444 publications were selected for data extraction (see Supplementary Table 1).

**Figure 2:**
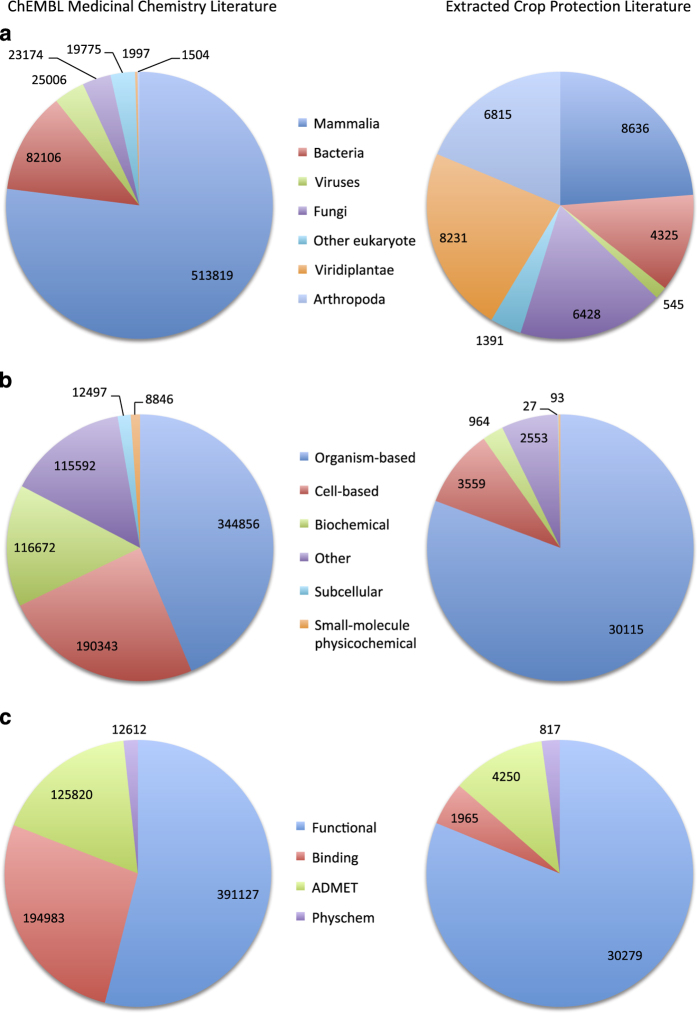
Comparison of crop protection and medicinal chemistry data sets. Pie charts showing a comparison of the features of the extracted crop protection assays with existing ChEMBL data (medicinal chemistry literature): (**a**) target organism distribution by number of assays, (**b**) assay format distribution by number of assays, (**c**) assay type distribution by number of assays.

This yielded a data set of 40,261 compound records, 37,311 assays (see Supplementary Table 2) and 245,370 bioactivity measurements. Of the compounds that were identified, 28,109 had structures that were not previously present in the ChEMBL database, indicating significant novelty compared with the standard medicinal chemistry content. Due to the complete inclusion of the Medicinal Chemistry Research journal, some extracted assays related to human health. However the vast majority of the assays measured herbicidal, fungicidal or insecticidal activity. Fig. 2 shows the distribution of target organisms, assay format and assay type across this data set, showing a distinct difference from the existing content of the database, particularly with respect to the proportion of the crop protection literature that represents organism-level phenotypic measurements rather than protein-based binding data.

Data were deposited in the ChEMBL database (version 19, released 23rd July 2014; Data Citation 1) and are accessible via a web-interface (https://www.ebi.ac.uk/chembl/), web-services (https://www.ebi.ac.uk/chembl/ws), and in a variety of download formats (ftp://ftp.ebi.ac.uk/pub/databases/chembl/ChEMBLdb/ and ftp://ftp.ebi.ac.uk/pub/databases/chembl/ChEMBL-RDF/). Since ChEMBL and PubChem BioAssay regularly exchange data, the data set is also available via PubChem (https://www.ncbi.nlm.nih.gov/pcassay).

## Technical Validation

While all data within the set were extracted from peer-reviewed scientific publications, there is always a possibility of errors being introduced, either by the original author or by the manual data extraction process. For this reason, additional data curation and validation was carried out (see methods). In particular, assay descriptions and target assignments were checked and corrected by a second curator, and chemical structures were checked for chemistry errors (such as incorrect valence) and standardized. An automated process was used to detect potential errors in activity values or their units. For example, an IC50 value with units of ml would be flagged with the data_validity_comment ‘Non standard units for type’, while a Ki value of 7.4 M would be flagged as ‘Outside typical range’.

Once released, data within ChEMBL are further checked and corrected on an ongoing basis. Therefore any additional errors or inconsistencies detected within the crop protection data set (either following feedback from users, or in response to our own error detection processes) will be corrected in subsequent releases. However, the data will still remain available in its original form, as released in ChEMBL_19, from the FTP site.

## Usage Notes

The ChEMBL web interface (https://www.ebi.ac.uk/chembl/) provides a number of mechanisms for searching and retrieval of relevant information. Target information in the database is classified both in terms of protein family but also by species. Using the ‘Browse Targets’ tab and switching to the ‘Taxonomy Tree’ view therefore allows users to retrieve all targets (both protein and non-molecular or species-level targets) belonging to a particular kingdom or phylum (e.g., Viridiplantae, Arthropoda, Fungi). Members of the resulting target list can then be selected/deselected and bioactivity data be retrieved or filtered using a drop-down menu. Alternatively, a keyword search is available to search across compound and target names and synonyms, assay descriptions and document titles/abstracts. Therefore entering a search term such as ‘herbicidal’ into the search box and selecting ‘Assays’ will retrieve a list of all assay descriptions containing this keyword. The ChEMBL document identifiers listed in Supplementary Table 1 can also be entered into this search field in order to return all data associated with that document. Finally, users can retrieve compounds similar to a structure of interest (e.g., a known pesticide) using the ‘Ligand Search’ tab, which provides identity, substructure and similarity searching functionality. Further details of the ChEMBL interface and its functionality are provided in previous publications^1,2^ and on the FAQ page (https://www.ebi.ac.uk/chembl/faq).

While the web interface provides the basic functionality to interrogate the data relating to a particular compound, target or species of interest, users wishing to perform large-scale data retrieval or analysis may wish to instead use the ChEMBL web services or download a version of the database for local installation. The ChEMBL web services homepage (https://www.ebi.ac.uk/chembl/ws) provides information on the web service calls available and an example Python client. Similarly, schema documentation (including a schema diagram) is provided alongside the various download formats on the FTP site, and example SQL queries are provided on the ChEMBL FAQ page. Both the ChEMBL interface and web services are provided over a secure HTTPS connection. Alternatively, a local installation of the myChEMBL virtual machine provides local access to the full ChEMBL database along with a plethora of computational tools and examples for data analysis^42^. Other open-source tools such as Open Babel^43^ or RDKit^44^ can also be used to compare and analyze compound structures, using the structure-data file provided on the FTP site.

Users should always be aware that although data are extracted manually and further curated, some errors are inevitable in such a large data set and therefore data should always be treated with caution. For example, upon identifying an interesting activity data point for a compound or target of interest, it is always prudent to consult the original publication to ascertain further details of the experimental procedures before using the data as the basis for further experiments. Similarly, for large-scale applications such as the construction of target prediction models, it is advisable to carefully filter the data to remove potential duplicates or erroneous values (for example using the data_validity_comments)^45^ and to pay attention to the details of the assigned target. For example, the target type of ‘PROTEIN FAMILY’ usually denotes a non-subtype specific assay and may not be appropriate for inclusion, similarly the relationship_type flag indicates whether the target mapped is the exact target used in the assay.

## Additional Information

**How to cite this article:** Gaulton, A. *et al.* A large-scale crop protection bioassay data set. *Sci. Data* 2:150032 doi: 10.1038/sdata.2015.32 (2015).

## Supplementary Material



Supplementary Table 1

Supplementary Table 2

Supplementary File 1
